# Protocol for evaluating the impact of a national school policy on physical activity levels in Danish children and adolescents: the PHASAR study - a natural experiment

**DOI:** 10.1186/s12889-018-6144-8

**Published:** 2018-11-08

**Authors:** Natascha Holbæk Pedersen, Sofie Koch, Kristian Traberg Larsen, Peter Lund Kristensen, Jens Troelsen, Niels Christian Møller, Jan Christian Brønd, Jacob von Bornemann Hjelmborg, Søren Brage, Anders Grøntved

**Affiliations:** 1 Centre of Research in Childhood Health, Research Unit for Exercise Epidemiology, Department of Sports Science and Clinical Biomechanics, University of Southern Denmark, Campusvej 55, 5230 Odense M, Denmark; 2 Research unit for Active Living, Department of Sports Science and Clinical Biomechanics, University of Southern Denmark, Campusvej 55, 5230 Odense M, Denmark; 3 The Danish Twin Registry, Epidemiology, Biostatistics and Biodemography, SDU eScience Centre, J. B. Winsløws Vej 9, 5000 Odense C, Denmark; 4 MRC Epidemiology Unit, Cambridge School of Clinical Medicine, Institute of Metabolic Science, University of Cambridge, Box 285, Cambridge Biomedical Campus, Cambridge, CB2 0QQ UK

**Keywords:** Physical activity, School children, Accelerometry, Body mass index, Implementation, Policy

## Abstract

**Background:**

In 2014 the Danish Government introduced a wide-ranging school reform that applies to all public schools in Denmark. The reform involves changes in several aspects of the school structure and content. In a physical activity promotion perspective, a distinctive feature of the school reform is that it has become mandatory to integrate an average of 45 min of daily physical activity in the regular school day. The overarching objective of the PHASAR study is to evaluate the implementation and effect of this ambitious policy-driven physical activity promotion initiative on physical activity and overweight. This paper describes in detail the study protocol.

**Methods:**

The evaluation is divided into a quantitative effect evaluation and a combined quantitative and qualitative process evaluation. A total of 31 schools are enrolled in the PHASAR study including more than 2,000 school-aged children. Objectively measured physical activity data are obtained in the PHASAR study in 2017/18 and compared to repeated cross sectional data collected in four historical school-based studies from 1998 to 2012. Body mass index data from 2012 to 2018 will be collected from The Child Database, which includes repeated cross-sectional assessments on approximately 100,000 children annually. In the absence of a control group, interrupted time-series analysis will be used to evaluate pre- and post-reform physical activity and body mass index levels and trends. A characterization of the school environment for physical activity promotion on a political, environmental, organizational and individual level and school implementation processes will be conducted to evaluate the implementation process. Data will be collected using interviews, surveys, document analyses and observations.

**Discussion:**

The PHASAR study is a rare opportunity to evaluate the effectiveness of a nation-wide policy-driven school-based physical activity promotion initiative. The use of objectively measured pre- and post-reform physical activity and body mass index data combined with a characterization of the school implementation processes for physical activity promotion will provide a comprehensive source to evaluate the school reform. The study findings have the potential to influence national and international policy makers, health professionals and school staff.

## Background

Physical inactivity is a global public health threat causing more than 3 million preventable deaths yearly [1, 2]. The health benefits associated with regular engagement in physical activity (PA) among children and adolescents include improved cardiorespiratory- and muscular fitness, bone health, body composition, and cardiovascular- and metabolic risk factor levels. In addition, benefits of PA also comprise mental aspects of health such as improved physical self-perceptions and enhanced self-esteem [3]. Despite the realization of the importance of PA, young peoples’ and adults’ lifestyle have changed over the last several decades in most societies around the world. Today a large proportion of children and adolescents are not sufficiently physically active to achieve optimal health benefits [4]. These changes have been accompanied by rising trends in obesity; from 1975 to 2016, children’s and adolescents’ age-standardized mean body mass index (BMI) increased globally and in most regions [5].

School-aged children spend 30–35 h per week in school corresponding to approximately 40% of their waking hours [6, 7]. In Denmark, public (state) schools are free of charge and mandatory for children between 6 and 16 years of age, who do not attend private schools; therefore children from all social classes are numerously represented in this setting [8]. Moreover, schools have the potential to provide infrastructure, facilities, and staff to support PA promoting initiatives making these institutions a key setting for PA promotion [6, 7, 9]. Internationally, various interventions have been conducted to promote PA in schools [10–12]. These include providing extra physical education (PE) lessons, health education, after-school programs, parental engagement, newsletters, and sports equipment [10, 11]. Previous efforts have been unilateral and there is a need for intervention at all levels to target insufficient levels of PA [13].

**Fig. 1:**
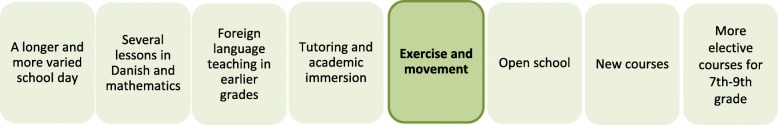
Elements of the Danish school reform. A total of eight elements were introduced in the 2014 school reform; exercise and movement was one of them. Open school is an element of the reform that focus’ on cooperation with the local community, e.g. sports associations, companies or museums

In 2014 the Danish Government introduced a new and unique legislative PA promoting initiative as a part of a national school reform (Fig. 1). The school reform was introduced in all municipal primary and lower secondary public schools. The school reform entailed a longer and more varied school day intending to alternate between regular classes and activities such as play, movement, projects, and workshops [14]. The overall aim of the school reform was to ensure that all children meet their full learning potential in order to counterbalance social background impacting academic performance, and to ensure well-being among all children and adolescents attending public schools [14]. In a PA promoting perspective, a distinctive feature of the school reform is the incorporation of 45 min of PA and movement in the regular school day [14].

From 2011 and onwards it has been compulsory for school nurses to report height and weight on school-children to *The Child Database* (In Danish: *Børnedatabasen*) making it possible to evaluate the effect of increased PA in schools on body composition. No corresponding national routine surveillance of objectively measured PA exists. At University of Southern Denmark, however, objectively measured PA data have been collected in several population-based school-based research projects since 1998 and onwards providing pre-reform PA data. This PA data, *The Child Database* and the introduction of the Danish school reform provide a unique opportunity for evaluating the implementation and effects of a nationwide school-based health initiative on PA and body composition, which is the overarching aim of the *Physical Activity in Schools After the Reform (PHASAR) study*. The objective of the present paper is to present the study protocol of the PHASAR study.

## Methods

### Study design and objectives

An effect evaluation of a nation-wide policy presents several scientific challenges, as it is impossible to have a randomized comparison group or to undertake comparison to a matched contemporary control group. As a consequence, bias and confounding may threaten the validity of the causal inference attributing any observed differences to the policy change alone. In the absence of a contemporary comparison group, a quasi-experimental design using interrupted time-series (ITS) analysis [15] is a valuable alternative that has been used previously to examine the effectiveness of nation-wide policy changes [16–19]. A basic pre-post analysis based on one assessment before and after the reform would fail to account for any trends in PA and BMI before the reform.

The evaluation will be based on routine-collected health data, historic- and newly collected researcher-led population-based quantitative data, and a mixed-methodology part that also include gathering of qualitative data. Thus, the evaluation consists of two overall parts: a quantitative effect evaluation and a combined quantitative and qualitative process evaluation. The effect evaluation is conducted to examine the effect of the school reform on total-; school- and leisure time PA and BMI. The process evaluation is conducted to obtain an understanding of the schools’ implementation processes, and to examine how different school characteristics potentially may influence PA levels of school-aged children.

### Recruitment and participants

**Fig. 2:**
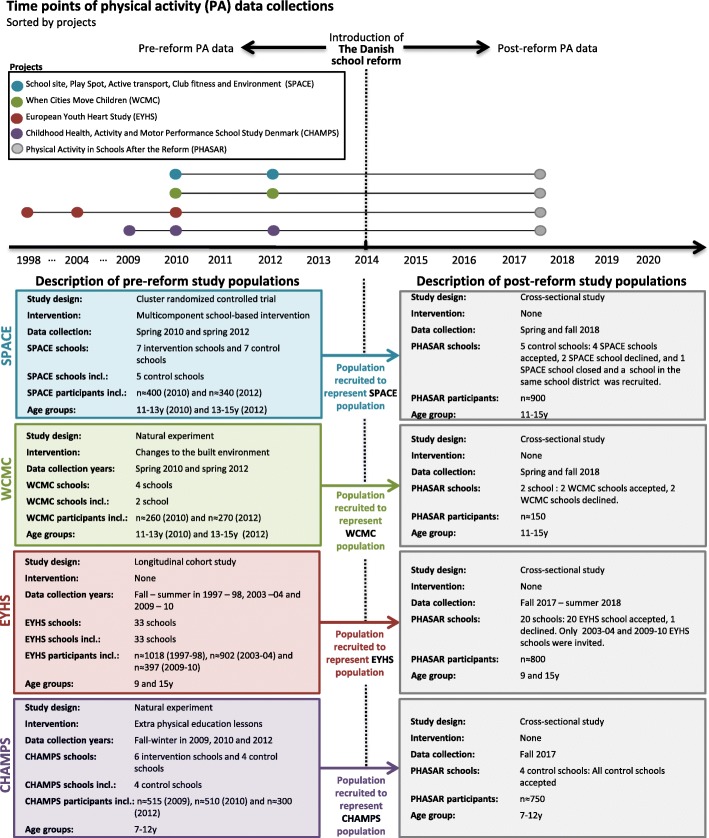
Time points of physical activity data collections and description of study populations. Please notice that population sizes are merely estimates. SPACE: School site, Play Spot, Active transport, Club fitness and Environment . WCMC: When Cities Move Children. EYHS: European Youth Heart Study. CHAMPS: Childhood Health, Activity and Motor Performance School Study Denmark

To be able to evaluate the effects of the Danish public school reform on PA levels it is imperative to have repeated cross-sectional data before (pre-reform) and after (post-reform) the introduction of the reform. PA data deriving from four historical studies completed from 1998 to 2012 are used to evaluate pre-reform PA levels and trends. PA data collected from August 2017 – September 2018 are used to evaluate post-reform PA levels (Fig. 2).

#### Pre-reform study populations

As previously stated, there are no routine surveillance systems of PA in Danish school children; therefore, we identified individual studies with information on these variables. The validity of self-report as a sole source of information is limited in this age group and may potentially also drift over time, which would defeat the purpose of our investigation [20]. Thus, objectively measured PA data collected in four school-based studies before 2014 comprise the historical data used to evaluate pre-reform PA levels and trends. The four studies are 1) The European Youth Heart Study (EYHS) conducted in 1997–98, 2003–04 and 2009–10, 2) When Cities Move Children (WCMC) conducted in 2010 and 2012, 3) Childhood Health, Activity, and Motor Performance School Study Denmark (CHAMPS-DK) conducted in 2009, 2010 and 2012, and 4) School site, Play Spot, Active transport, Club fitness and Environment (SPACE) conducted in 2010 and 2012. Accelerometry was used to assess PA in all four studies. Sampling and recruitment of children and adolescents included in these four studies have been described elsewhere [21–24]. These studies comprise PA data on approximately *n* = 2,500 1st to 9th grade children from 44 schools in 7 municipalities in the Region of Southern Denmark and the Capital Region of Denmark. An overview of the historical studies and study populations is presented in Fig. 2.

#### Post-reform study population

To enable evaluation of post-reform PA levels it is imperative to conduct an additional data collection after the introduction of the school reform in 2014. Thus, a new cross-sectional data collection was initiated in 2017 including school-aged children and adolescents that broadly represent the source population of the four historical studies (Fig. 2). Consequently, the same schools and age-groups as in the four historical studies were invited to participate in the PHASAR study. Two of the historical studies (CHAMPS-DK and SPACE) introduced PA promoting initiatives as part of their study; to minimize any influence from participation in these interventions we chose only to include and recruit control schools from these studies (Fig. 2). The schools were contacted continually in 2017 and invited to participate in the study. A total of 36 schools were invited in 2017 and 31 schools accepted the invitation to participate.

All parents or guardians of the invited children received an invitation that explained the objective, content and procedures of the study. Children received similar oral information at the schools provided by the research team. If parents or guardians, or the child, did not want to participate, they were able to withdraw at any stage. A child or an adolescent was found eligible to participate if 1) the child attended one of the public schools and age groups (grades) that had already been included in one of the four pre-reform studies, and 2) the child did not suffer from any physical disabilities or injuries preventing physical activity.

### Study procedures

**Fig. 3:**
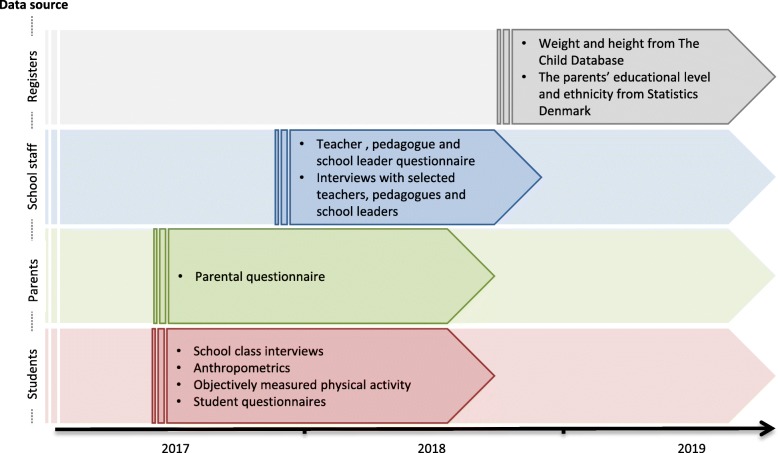
Overview of the post-reform data collection period

Post-reform data were collected at the schools from August 2017 till September 2018. Since PA levels in Danish school children are characterized by seasonal variation it was important to take such variation into account [25]. Consequently, the post-reform data collection was matched for season to their respective historical sources when possible. PA data matching the sampling of the EYHS population were collected during the entire school year, data matching the CHAMPS population were collected during fall/winter, and data matching the SPACE and WCMC population were collected during spring and fall. An overview of the 2017/18 data collection period is presented in Fig. 3. Standardized testing protocols were made to ensure data quality, and trained research assistants collected all data.

Prior to the initiation of the PHASAR study a pilot study was conducted to optimize all study procedures. Three school classes from one private school participated in the pilot study (*n* = 55).

### Effect evaluation

Data used to evaluate the effect of the school reform on PA and BMI levels are described in the sections below.

#### Physical activity

PA is assessed objectively by waist-mounted accelerometry. ActiGraph monitors (am7164, gt1m, and gt3x) were used in the four historical studies and Axivity AX3 monitors are used in the present study. The ActiGraph monitors have previously been validated in both children and adolescents against various criteria and have been found to be a valid method (*r* = 0.54–0.66) to assess habitual PA levels [26, 27]. All monitors measure acceleration; however, the only output available with devices used in the four historical studies is counts per unit time, whereas Axivity AX3 monitors store raw acceleration data in g, typically in 100 Hz resolution. ActiGraph counts can be generated from Axivity AX3 raw acceleration by exporting the data into the ActiLife gt3x binary files and process the files in ActiLife. The ActiLife gt3x binary files generated from the Axivity AX3 raw acceleration use a 30 Hz sampling frequency to avoid the known bias observed with sampling frequencies like the 100 Hz [28]. The OmGUI software available with the Axivity AX3 device is used to down-sample data into 30 Hz sampling frequency and a special in-house software is used to export the raw acceleration into the gt3x binary files. The integrity of the data files was evaluated by comparing the sample-by-sample raw acceleration stored in the Axivity AX3 data files with raw acceleration exported into a CSV file using the ActiLife software. The in-house developed software Propero is used for the final data reduction and quality control.

#### Body mass index

**Fig. 4:**
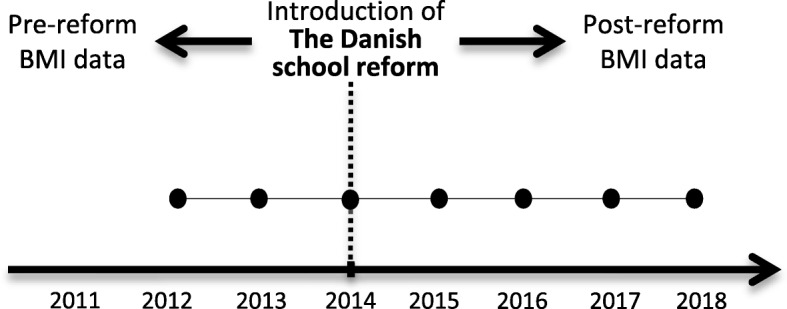
Time points of body mass index data collections. Data is collected via The Child Database

BMI calculated as weight divided by height-squared is collected via the nationwide surveillance database *The Child Database*. In Denmark, all school-aged children are offered a preventive health examination obtained by either school nurses or medical doctors according to law. In 2011, it became compulsory for all Danish municipalities to annually report data on height and weight to *The Child Database* via the Danish Board of Health Electronic Report System. Data are considered complete from 2012 and onwards, and from this time data on approximately 100,000 children aged 6–7 years (pre-preparatory classes), 9–13 years (intermediate classes) and 14–15 years (lower secondary education) are available annually. In the pre-preparatory classes the first assessment obtained is reported to *The Child Database*. If several measurements are conducted during the intermediate classes the measurements obtained closest to the child’s 11th birthday will be reported. In the lower secondary education classes the last measurement obtained before the child finishes school will be reported. Data from 2012 to 2018 will be extracted from *The Child Database* in 2019 (Fig. 4).

In addition, height, weight and waist circumference are assessed on the study population included in the PHASAR study data collection in 2017/18 (Fig. 2). These anthropometric data were also collected in all four historical studies. Thus, it is possible to use the body composition data for the purpose of providing descriptive data and stratification when examining PA before and after the introduction of the school reform.

#### National registers

For all participants civil registration number is collected making it possible to extract additional data from national registers (ethnicity, socioeconomic status etc.). These additional data will be useful for stratification purposes and in minimizing selection bias (if necessary).

### Process evaluation

Data used to complete the process evaluation and to evaluate the implementation of the school reform are described in the sections below. The process evaluation is primarily based on post-reform data. However, some historical survey data are included to compare pre- and post-reform data.

#### Questionnaires

Four different questionnaires were completed: a student questionnaire, parental questionnaire and one for school teachers and pedagogues, and one for the school management. All questionnaires were completed electronically.

#### Student questionnaire

Children in 5th–9th grade were asked to complete the questionnaire. The student questionnaire contained questions on leisure-time PA, sedentary behavior, transportation habits, living conditions, school satisfaction, well-being, screen time, sleep duration and assessment of neighborhood characteristics (physical environment, facilities, access to facilities etc.), and conditions for being physically active in school and the surrounding neighborhood. Some of the question items have previously been used in the historical studies and other national and international surveys concerning children’s PA level and health.

The children completed the questionnaire on tablets during a school lesson (45 min) the last day of wearing the accelerometer. Two or three trained research assistants were present during the session and instructed the children in how to complete and access the questionnaire using unique ID-numbers. Research assistants assisted if any questions arose during the questionnaire. If one or more children had difficulties reading and understanding the questions a research assistant assisted these children in a separate room. All children were instructed to complete the questionnaire individually without talking to classmates.

#### Parental questionnaire

All parents or guardians were asked to complete a questionnaire containing questions on living conditions, educational level, assessment of neighborhood, possibilities for being physically active in their neighborhood and children’s leisure time activities. Moreover, parents of children in 1st to 4th grade were asked to answer questions containing facilities in their neighborhood, access to facilities, children’s transportation habits and children’s sleep duration.

Only one parent was asked to complete the questionnaire. After the data collection at the school the school administration sent out the questionnaire link to the parents. An estimated 15–20 min were projected to complete the questionnaire. To increase the response rate reminders were sent out twice via schools’ parental email list to those parents who lacked filling out the questionnaire.

#### Teacher questionnaire

Danish-, math-, English- and assistant teachers from participating classes were asked to complete a questionnaire concerning their education level, time of employment, use of PA when teaching, kinds of physical activities used in classroom teaching, school’s physical environment, use of open school, their PA level and transportation habits. Teachers were asked to complete the questionnaire immediately after the data collection at the school. The completion took approximately 20 min.

#### School management questionnaire

The questionnaire for school management included questions on school’s physical environment, school facilities for PA, school policies regarding PA and health, open school, initiatives for PA and general health for children, and questions on implementation of the ‘45 minutes of PA per day’ initiative. School principals or a similar administrative person with knowledge about the implementation process were asked to complete the questionnaire. The concerned respondent was asked to complete the questionnaire immediately after the data collection at the school. An estimated 45–60 min was projected to complete the questionnaire.

#### Health policies

The school administration was asked to forward relevant papers on health policies, schools principles or other papers relevant for identifying school’s implementation of 45 min PA per day.

#### Timetables

The administration was further asked to forward detailed timetables for the participating classes for the week when data collection was taking place. Timetables contributed information on organization of the school day for use in combination with the objective assessment of PA.

#### Interviews

To gain knowledge about the organization of PA during teaching, and to examine the type of PA that is practiced, all participating classes were asked to take part in a class-interview regarding physical activities during recess and lessons. The class-interviews lasted for approximately 10–15 min and were conducted during the first visit at the schools.

To gather in-depth data of the implementation of 45 min PA per day on a teacher- and school management level, 11 representative schools were selected for interviews. The interviews were semi-structured and were based on the Ecological framework for understanding effective implementation and the Practical, Robust Implementation and Sustainability Model (PRISM) as theoretical starting point [29, 30]. The interview included identification and assessment of specific school-based PA programs, school’s physical environment for PA and health policies. Moreover, interviews were used to get in-depth data on the organization of non-scheduled PA, integration of PA during classroom teaching, and as a tool for identifying differences in health policies and PA programs across different tiers. One school representative from each school with in-depth knowledge of the implementation process was asked to participate. The interviews lasted for approximately 60 min.

#### Observational data

To detect and evaluate the built environment as a determinant for PA, observational data were collected at the schools that participated in the interviews. The built environment was evaluated using two measures: the surface of each school playground (m^2^) and the number of permanent play facilities. The area of the schoolground was mapped in detail using Geographic Information System (GIS). GIS was further used to map school vegetation, open green spaces and outdoor play facilities [31–34]. Moreover, GIS was used to examine access to open public spaces (parks, green areas), street patterns and vegetation in the school neighborhood [31]. Inspired by Nielsen et. Al (2010) all facilities for PA were registrated. Facilities were counted as physical structures if they previously had been observed to be used by the children for play and/or sports activities during recess [35]. The number of facilities at each school was counted twice during the day of observation while the children were at play [35].

### Statistical methods

Descriptives of the cohort will be obtained for characterization and representativeness. Interrupted time-series (ITS) analysis will be used as the overall statistical method. Based on at least two repeated cross-sectional assessments before and after the introduction of the school reform, ITS estimates the pre-reform slope, the change in level around the time of introduction of the reform accounting for the pre-reform trend, and the change in slope after the reform. The results of the change in level and the change in slope represent the estimates of the effect of the reform assuming a linear before- and after time trend. With regard to BMI, *The Child Database* provides sufficient pre- and post-reform data points to investigate trends. With respect to PA, pre-and post reform analysis will be conducted.

Multi-level regression and significance tests will be utilized for analyses in both parts of the study (the effect and process evaluation), as qualitative analyses will be used for analyses with respect to the process evaluation.

### Justification of sample size

With n ≈ 100,000 in each BMI time point before and after the school reform, we have 80% power (alpha level = 0.05) to detect a difference of 0.04 BMI points assuming a standard deviation of 3.2 BMI points (data from EYHS). With n ≈ 2,000 in each PA time point before and after the reform, we have 80% power (alpha level = 0.05) to detect a difference in moderate-vigorous PA of approximately 2 min/day assuming a standard deviation of 24 min/day (data from SPACE).

### Reporting statements

The REporting of studies Conducted using Observational Routinely-collected health Data statement (RECORD) will be used when reporting results from the effect evaluation. Cochrane Qualitative and Implementation Methods Group guidance series—paper 6: reporting guidelines for qualitative, implementation, and process evaluation evidence syntheses will be used to report results with respect to the process evaluation.

## Discussion

The public school reform implemented in the Autumn of 2014 by the Danish government provides a unique opportunity for evaluating the effectiveness of this ambitious nation-wide policy-driven school-based PA promotion initiative. By international comparison, the magnitude of the strengthened focus on PA as a key component of public school life is exceptional. Access to historical data on objectively measured PA and body composition provides a rare opportunity to evaluate the effect of this ambitious legislation on PA- and BMI levels in school-aged children and adolescents. Further, in combination with the process evaluation the study aims to understand the different implementation processes and thereby the expected variation in uptake of the reform on PA. As mentioned earlier, several randomized controlled studies have evaluated the effect of various school-based PA promotion interventions [11]. Because many randomized controlled studies evaluating the effectiveness of school-based PA have been researcher-led interventions and typically conducted in non-representative samples they often have poor external validity. When possible, complementing any evidence on effectiveness from randomized controlled studies with that from observations based on natural experiments are therefore highly valuable. Thus, the present project will significantly advance and complement the evidence on the effectiveness of school-based PA interventions. The school-level data characterizing how individual schools have implemented the PA requirement prompted by the reform will be an important resource to support and help explain findings in the effectiveness analysis, including variations in effect. Furthermore, these observations can be used to establish a number of ‘best practice’ examples, which are important for the government, municipalities, schools and other non-governmental organizations working with promotion of PA in schools.

To our knowledge the Danish school reform is one of the first policy-driven initiatives aiming to integrate a substantial amount of PA in the regular school day. In 2018, similar policies will be introduced in the Norwegian and Taiwanese public schools aiming to implement an average of 60 min/day and at least 150 min/week, respectively [36, 37]. In Hungary, daily PE became part of the program of the Government in 2010. After its gradual implementation, from the school year 2015/16, all students of all grades in primary and secondary school were supposed to take part in 5 PE classes per week, each lasting 45 min [38]. In Scotland, the PA programme The Daily Mile is being promoted by the the Scottish Government. The programme aims to get the students outside to run for 15 min (1 mile) during class each day. An evaluation of the programme found it to be effective in improving MVPA, cardiorespiratory fitness and body composition [39]. Similar policy-driven initiatives have been introduced in some of the provinces and states in Canada and in the U.S. [9, 40–43]. However, none of these policies have been initiated on a national political level. To our knowledge, only a few evaluations of these PA promoting initiatives have been conducted - primarily on the initiatives in Canada. A growing number of jurisdictions in Canada have adopted the Daily Physical Activity (DPA) guidelines that increase the requirement for PA in schools [40, 44]. In Ontario, Canada, DPA guidelines stipulate that all school boards ensure that students attending 1st to 8th grade participate in a minimum of 20 min of sustained MVPA each day during instructional time [45, 46]. In a cross-sectional study, Stone et al. [45] examined the proportion of schools in Ontario that participated in DPA and the proportion that actually met the guidelines of 20 min of MVPA a day assessed using accelerometry. Results showed that the majority of children did not meet the required frequency or intensity of the DPA policy indicating that the DPA guidelines were not fully implemented at the included schools [45]. An additional cross-sectional study conducted by Allison et al. [46] evaluated the implementation fidelity to the DPA guidelines. The results concurred with the results by Stone et al. [45] as they concluded the implementation of the guidelines to be incomplete. None of the above-mentioned studies had access to PA data before the introduction of the DPA guidelines making it impossible to conclude on the effect of the initiative. Consequently, the effect of policy-driven PA promotion initiatives, similar to the school reform in Denmark, is still unknown. To our knowledge, the present study will be the first study to examine the implementation and effects of a nationwide policy aiming to promote PA in public schools.

## Strengths and limitations

### Study design

Conducting a randomized controlled trial (RCT) to evaluate the nationwide school reform is not possible in the present context, since the school reform is a policy that applies to all public schools across Denmark (*N* = 1,267). Thus, it is not possible to have a randomized comparison group or to compare with a contemporary matched control group to prevent bias and confounding, which is a limitation of the current study [47, 48]. However, in the absence of a control group, the interrupted time-series (ITS) analysis is a statistical method that serves to mimic the control group, i.e. by statistical matching of other parameters and treating time and change time-points as exposure variables. This technique has previously been utilized to examine the effectiveness of nation-wide policy changes [15, 16, 18]. Thus, this statistical method will be utilized to evaluate pre- and post-reform PA and BMI levels and trends.

Data is combined by pre-reform and post-reform PA data. To enhance the comparability of the new data collection with the historical source population and to minimize potential confounding, children with the same age will be recruited at the same schools as in the historical studies. Thus, the recruitment is limited to the schools represented in the four historical studies. Since the school reform has been introduced in all public schools in Denmark, the study population in the present study should represent the target population, which under optimal conditions would be all children and adolescents in Denmark. All historical studies aimed to be representative only for a predetermined community and not the whole country of Denmark. The aggregation of the four samples does, however, improve the representativeness on a national level, but it is still only a minority of all municipalities in Denmark that are represented in the historical and new samples. This likely will affect the external validity of the present study with respect to the investigation of PA levels, in contrast to the body composition data which largely represent all children from Denmark.

### Study population

The main reason for only including control schools of the historical intervention studies (Fig. 2) was to increase the internal validity of our results by minimizing potential localized intervention effects. Thirty-six schools were invited to participate in the present study. During the recruitment process five schools declined to participate, and a total of 31 schools were enrolled. Unfortunately, most of the schools that declined were geographically located in the same parts of Denmark, which further reduced the geographical representativeness.

Using a mixed methods approach that includes qualitative and quantitative methods strengthens the study, since the different types of data complement each other [49]. A process evaluation including different types of data should help understand and explain the findings of the effect evaluation. Moreover, observations from the present study may be able to identify a number of “best practice” examples, which would be highly valuable for the government, municipalities, schools and other non-governmental organizations concerned with promotion of PA in schools. However, it is important to emphasize that the 45 min of daily PA in schools are merely a single fragment of an ambitious and comprehensive school reform (Fig. 1). As previously mentioned the Danish school reform consists of eight initiatives that every school must adhere to. Thus, it will be wrong to conclude that any change in PA solely is a result of the requirements of 45 min of daily PA; rather, any changes in PA levels could be a result of the entire policy implementation.

The PHASAR study provides a rare opportunity to evaluate the effectiveness of a nation-wide policy-driven school-based PA promotion initiative. The use of objectively measured pre- and post-reform PA and body composition, and the characterization of school implementation processes for PA promotion on a political, environmental, organizational and individual level will provide a comprehensive source to evaluate the school reform. Therefore, the study findings have the potential to influence policy makers, health professionals and school staff.

## Abbreviations

BMI: Body mass index
CHAMPS-DK: Childhood Health, Activity and Motor Performance School Study Denmark
DPA: Daily Physical Activity
EYHS: European Youth Heart Study
GIS: Geographic Information System
ITS: Interrupted time-series
MVPA: Moderate to vigorous physical activity
PA: Physical activity
PE: Physical education
PHASAR: Physical Activity in Schools After the Reform
RCT: Randomized controlled trial
SPACE: School site, Play Spot, Active transport, Club fitness and Environment
WCMC: When Cities Move Children
